# British Lying-In Hospital

**Published:** 1902-04-12

**Authors:** 


### BRITISH LYING-IN HOSPITAL.

ELEVEN gentlemen, four of whom were members of the Board of Management, responded to the secretary's notice of the annual general meeting, fixed for five o'clock on Monday, March 24th. Mr. C. E. FARMER, Chairman of the Board, presided, and moved the adoption of the report and accounts.

THE PATIENTS INCREASE.

Last year, said Mr. Farmer, there were 113 more patients than the year before, namely, 467; and 616 out-patients were delivered, or 87 more than in the previous year. There was not a single maternal death, which said a good deal for the care and skill of the matron and her assistants.

THE NURSES' HOME.

The speaker reminded those present that a scheme was passed by a special meeting not many weeks ago which authorised the spending of £7,000 on a Nurses' Home. The contract, he said, would be signed probably in a week's time, and then No. 28 in Bellerton Street would be pulled down and the Home built on its site. He hoped it would be completed early in 1903. Having regard to the large sums other hospitals asked for and got, he thought £7,000 was a very modest request. He urged the governors to make the hospital's wants known to their friends, so that donations might be received. The Home would accommodate 28 nurses, and rooms would be set free so as to take in 10 more patients; this would make a difference of not less than 200 patients in the course of a year.

SUBSCRIPTIONS IN ARREARS.

Again the CHAIRMAN had to call attention to the fact that subscriptions to the amount of £47 15s. had only been paid after the closing of the accounts. Last year he said he hoped this item might be eliminated from the balance-sheet; this hope had not been fulfilled, though the amount in arrear was very much smaller this time.

Sir Frederick Treves was unanimously elected consulting surgeon in place of the late Sir William MacCormac, Bart., and after the re-election of officers and the usual votes of thanks, the meeting ended, having lasted about a quarter of an hour.

In the course of his remarks, Mr. FARMER said, "We have the best of matrons and everything is done in the best possible way for the benefit of the patients and of the hospital. Miss Knott, the new matron, was assistant matron for three and a half years under Miss Cook, whose death, rather more than a year ago was caused by blood poisoning contracted during the performance of her duties. Miss Knott was temporarily appointed in her place, and last August the appointment was made a permanent one. She was trained at Pendlebury (Children) and Guy's (General) before taking up the midwifery course. There have been one hundred cases in the hospital since the beginning of the present year, and for none of these has it been necessary to call in medical aid. The Nurses' Home, it may be added, is a real need the present accommodation is very unsatisfactory, and moreover, some of the nurses have to sleep at a house in Little Russell Street, which, though very comfortable and well managed, is too far away. All who have to do with the hospital will be thankful when the new Home is built and ready for occupation, and that it is a worthy object is proved by the fact that King Edward's Fund has given a special grant of £200 towards it. The total ordinary income during the year was £2,637 15s. 5d., and the total ordinary expenditure was £2,472 11s.

NOTE.

It may be pointed out that though the number of new beds to be made available for patients is only 10, this is 50 per cent. of the present number, which does not exceed 20. It is possible that the difference on the year's total may be found to exceed 200, the Chairman's estimate; for, with an additional ward, cleaning will be made easier, only one ward being necessarily closed at a time; the length of stay, moreover, is only 14 days at this hospital.

